# A microarray analysis of gene expression patterns during early phases of newt lens regeneration

**Published:** 2013-01-28

**Authors:** Konstantinos Sousounis, Christian S. Michel, Marc Bruckskotten, Nobuyasu Maki, Thilo Borchardt, Thomas Braun, Mario Looso, Panagiotis A. Tsonis

**Affiliations:** 1 Department of Biology and Center for Tissue Regeneration and Engineering at Dayton, University of Dayton, OH; 2 Max-Planck-Institute for Heart and Lung Research, Ludwigstrasse 43, 61231 Bad Nauheim, Germany

## Abstract

**Purpose:**

*Notophthalmus viridescens*, the red-spotted newt, possesses tremendous regenerative capabilities. Among the tissues and organs newts can regenerate, the lens is regenerated via transdifferentiation of the pigment epithelial cells of the dorsal iris, following complete removal (lentectomy). Under normal conditions, the same cells from the ventral iris are not capable of regenerating. This study aims to further understand the initial signals of lens regeneration.

**Methods:**

We performed microarray analysis using RNA from a dorsal or ventral iris isolated 1, 3, and 5 days after lentectomy and compared to RNA isolated from an intact iris. This analysis was supported with quantitative real-time polymerase chain reaction (qRT-PCR) of selected genes.

**Results:**

Microarrays showed 804 spots were differentially regulated 1, 3, and 5 days post-lentectomy in the dorsal and ventral iris. Functional annotation using Gene Ontology revealed interesting terms. Among them, factors related to cell cycle and DNA repair were mostly upregulated, in the microarray, 3 and 5 days post-lentectomy. qRT-PCR for rad1 and vascular endothelial growth factor receptor 1 showed upregulation for the dorsal iris 3 and 5 days post- lentectomy and for the ventral iris 5 days post-lentectomy. Rad1 was also upregulated twofold more in the dorsal iris than in the ventral iris 5 days post-lentectomy (p<0.001). Factors related to redox homeostasis were mostly upregulated in the microarray in all time points and samples. qRT-PCR for glutathione peroxidase 1 also showed upregulation in all time points for the ventral and dorsal iris. For the most part, mitochondrial enzymes were downregulated with the notable exception of cytochrome c–related oxidases that were mostly upregulated at all time points. qRT-PCR for cytochrome c oxidase subunit 2 showed upregulation especially 3 days post-lentectomy for the dorsal and ventral iris (p<0.001). Factors related to extracellular matrix and tissue remodeling showed mostly upregulation (except collagen I) for all time points and samples. qRT-PCR for stromelysin 1/2 alpha and avidin showed upregulation in all the time points for the dorsal and ventral iris.

**Conclusions:**

The results show that the dorsal iris and the ventral iris follow the same general pattern with some distinct differences especially 5 days after lentectomy. In addition, while the expression of genes involved in DNA repair, redox homeostasis, and tissue remodeling in preparation for proliferation and transdifferentiation is altered in the entire iris, the response is more prominent in the dorsal iris following lentectomy.

## Introduction

Whole organ regeneration is promising for medical applications. Many organ systems and models are being used for their ability to regenerate certain tissues and organs [1]. The red-spotted newt, *Notophthalmus viridescens*, has the ability to regenerate, after partial removal, many organs and tissues, including the heart [2], brain [3], tail, and limbs [4]. Newts also have the unique ability to regenerate whole organs such as the retina and lens of the eye even as adults. The lens is regenerated by transdifferentiation of pigmented epithelial cells (PECs) of the iris. Dorsal PECs are the only ones involved in lens regeneration while ventral PECs do not participate in the process. Dorsal and ventral PECs reenter the cell cycle at day 4 post-lentectomy, and by days 8–10, a dedifferentiated vesicle is formed in the dorsal iris. Thus, analysis of global gene expression in the dorsal and ventral iris could provide versatile answers why cells from one side can regenerate a lens and why the same cells from another side cannot. In addition, when individual genes were studied, important regulatory genes were expressed on the dorsal and ventral iris [5]. A small-scale microarray analysis using 373 genes showed similar expression pattern when examined at day 8 after lentectomy, which demarcates the stage of dedifferentiation and vesicle formation [6]. Congruent results were achieved by achieving microRNA expression data [7,8] and proteome analysis [9]. Consequently, our hypothesis is that the ventral iris initiates the process but is then inhibited. This is also implied by studies in which the ventral iris was induced to transdifferentiate by exogenous factors [10] or by studies in which gene expression-suppressive histone modifications were found specifically in the ventral iris [11].

To obtain a better idea of and new insights into this regulation, we examined gene expression patterns during the early phases of lens regeneration (1 day post-lentectomy to cell cycle reentry). We compared gene expression at day 0 (intact iris) with expression at days 1, 3, and 5 post-lentectomy. In the current study, we use custom-made microarrays generated from expressed sequence tags during heart regeneration [12]. Our data clearly indicate that the expression patterns between the dorsal and ventral iris are quite similar; however, there are distinct differences in the later stages (dorsal 5 days compared to ventral 5 days after lentectomy). Further, functional annotation and quantitative real-time polymerase chain reaction (qRT-PCR) analysis revealed that PECs prepare the ground for the main events of cell cycle reentry and for transdifferentiation even at the early stages.

## Methods

### Animals

Red-spotted newts, *Notophthalmus viridescens*, were obtained from Charles Sullivan Inc. Newt Farm (Nashville, TN). Procedures involving animals were approved by the Institutional Animal Care and Use Committee (IACUC) of the University of Dayton. Newts were anesthetized in 0.1% ethyl 3-aminobenzoate, methanesulfonic acid salt (MS222; Signa-Aldrich, St. Louis, MO) in phosphate buffer saline (PBS; 37 mM NaH_2_PO_4_ monohydrate, 58 mM Na_2_HPO_4_ anhydrous, pH 7.0) and then euthanized by decapitation.

### Lentectomy – sample preparation

Corneas were split between the dorsal and ventral area with a scalpel. Tweezers were used to pull the lens out. Both lenses were removed from each newt. Newts were then placed in appropriate humidified containers for 1, 3, or 5 days. At the desired time points, whole eyeballs were removed and placed in RNAlater Solution (Life technologies, Grand Island, NY). Subsequently the eyeballs were dissected to obtain the dorsal and the ventral irises, which were separated in different RNase-free microtubes (Fisher Scientific, Pittsburgh, PA) with RNAlater Solution.

### Microarrays

RNA pools from the samples were reverse transcribed to double-stranded cDNA using the SMART™ cDNA Library Construction Kit (Clontech, Mountain View, CA). This method allows amplification of double stranded full length cDNA by anchoring the 5' end of the isolated mRNA with a specific oligonucleotide [13]. Normalization of cDNA was performed based on DSN technology (Evrogen, Moscow, Russia). Cloned cDNAs were amplified to generate a library of more than 100,000 independent clones.

After plating, 100,000 individual clones were picked, and the inserts were amplified with PCR. After purification, inserts were resuspended in an appropriate spotting buffer (200 ng/µl PCR product in 3X SSC/1.5 M betaine) and spotted onto two sets of glass microarrays (Nexterion slides E, Schott). For each sample, 25 ventral or dorsal irises were pooled, and RNA was extracted with TRIzol (Life technologies). RNA was amplified with MessageAmp II aRNA Kit (Life technologies). For two-color microarray hybridization, cDNA labeling was performed with the SuperScript Plus Direct Labeling Kit (Life technologies), using Alexa 555 and Alexa 647 dye coupled nucleotides (Life technologies). Undamaged ventral or dorsal irises were compared with ventral or dorsal irises 1, 3, and 5 days after lentectomy. Two samples per time point were technically replicated by dye swap to reach n=4 per experiment. Scanning of microarrays was performed on a GenePix 4000B Microarray Scanner Molecular Devices LLC, Sunnyvale, CA) with GenePix Pro 6.0 Software, and data were statistically analyzed with Acuity 4.0 Software (Molecular Devices LLC). A complete list of all expressed expressed sequence tags (ESTs) during lens regeneration is available as a downloadable tab-delimited file. The links to these lists can be found at the bottom of each dorsal and ventral expression page (Expression search). Sequencing of the cDNA library and the sequence assembly are described in [12].

### Assembly of deregulated lens sequences

Sequences corresponding to 804 significantly regulated spots were assembled with SeqMan, version 8.0.2, with a minimum match percentage of 80% and a minimum sequence length of 100 bp, receiving 467 contigs with an average sequence length of 657 bp. For hierarchical clustering and heat map visualization, we used the Perseus software package. The expression values of the replicates were combined by mean. For visualization, we filtered the rows to have at least two valid values.

### Functional annotation

Assembled contig sequences were assigned to Gene Ontology (GO) terms by the BLAST2GO tool. For similarity searches, we used the NCBI NR database (blastx) with an e-value cutoff e^−10^ [14,15]. Contigs that were consistently up- or downregulated in all time points were obtained with perl scripts. A Venn diagram was created using VENNY.

### RNA extraction for quantitative real-time polymerase chain reaction

RNA extraction was performed following the TRIzol Reagent protocol (Life technologies). The tubes were centrifuged briefly, and RNAlater Solution was removed. For 20 iris pieces (10 newts; 20 irises), 500 µl of TRIzol Reagent were added, and using a pestle, the samples were homogenized and incubated at room temperature for 5 min. To eliminate pigments released to the solution, the samples were centrifuged for 10 min at 12,000 ×*g* at 4 °C. Briefly, chloroform was added, as the manufacturer suggests. From the aqueous phase, 200 µl were removed to limit the amount of DNA contamination. RNA precipitation was performed with 100% isopropanol, and RNA was washed with 75% ethanol. RNA resuspension was performed with RNase-free water. RNA quality and quantity were checked with a NanoDrop 2000 spectrophotometer (Thermo Scientific, Waltham, MA). A clear peak in the 260 nm and an A260/A280 ratio >2 indicated high-quality RNA for qRT-PCR. RNA used for this experiment was from different animals than the one used to probe the microarrays.

### Reverse transcription reaction

Reverse transcription (RT) reactions were performed with the First-strand cDNA Synthesis kit (GE Healthcare, Piscataway, NJ) following the recommended protocol for 1 µg total RNA and oligo(dT) primers for positive RT reactions and half the volumes for the negative RT reactions. For the negative RT reaction, no primers were added, and the samples were incubated for 5 min at 98 °C for enzyme inactivation before RNA was added. The success of the RT reaction was checked with polymerase chain reaction using TaKaRa Ex Taq (TaKaRa, Otsu, Shiga, Japan) and primers against *rpl27* and *gapdh* genes.

### Quantitative real-time polymerase chain reaction

qRT-PCR was performed in a Bio-Rad iCycler (Bio-Rad, Hercules, CA). qRT-PCR reactions were performed using iQ SYBR Green Supermix (Bio-Rad) and following the manufacturer’s protocol for a total volume of 25 µl. Melt curve analysis was performed for primer specificity. For each qRT-PCR run, a concentration gradient of the target gene cloned cDNAs was used. The concentration of the target genes in the samples was calculated using the standard curve made from the known concentration gradient (R^2^ >0.98) and the number of cycles (Ct). The concentration of the target gene in the samples was normalized against the housekeeping gene *rpl27* (6), and the relative expression level compared to the intact dorsal iris was calculated (day 0). All samples were run in triplicate. Statistical significance was calculated using the Student *t* test. Table 1 shows the primers that were used. All primers were tested for specificity in known newt sequences using the Basic Local Alignment Search Tool [16]. Annealing temperatures were checked by locating only the appropriate size band using polymerase chain reaction followed by agarose gel electrophoresis.

**Table 1:** List of primers for genes tested by qRT-PCR and annealing temperatures used for their respective target genes.

**Genes**	**Annealing temperature – band size**
**RPL27**	55 °C - ~100 bp
Forward 5′ – 3′	ATTTATGAAACCCGGGAAGG
Reverse 5′ – 3′	CCAGGGCATGACTGTAAGGT
**GAPDH**	55 °C – 509 bp
Forward 5′ – 3′	GCCTCCTGTACTACCAACTG
Reverse 5′ – 3′	CCCCACTCGTTGTCATACCA
**Cytochrome c oxidase subunit 2**	61 °C – 355 bp
Forward 5′ – 3′	ACACTAACGCAATAGACGCACAAGA
Reverse 5′ – 3′	ACGCCCATTGAGGGGACTGC
**Rad1**	61 °C – 341 bp
Forward 5′ – 3′	TGCGTGCCTCGACAACGTCC
Reverse 5′ – 3′	TCACCACCCCACCTTCCTCCA
**Stromelysin 1/2 a**	61 °C – 168 bp
Forward 5′ – 3′	GGGGGACAAAGACTCTCCCCGA
Reverse 5′ – 3′	CTGGTGTTCTTCAGTGTCCGGGT
**VEGFR1**	61 °C – 227 bp
Forward 5′ – 3′	TCCTGCAGCAGCCTGACCTTG
Reverse 5′ – 3′	GTTTGGGGCTGTGACTCGGC
**Avidin**	60 °C – 103 bp
Forward 5′ – 3′	TCGTTTCTCCTCTGACGGGCT
Reverse 5′ – 3′	TGCCCTGCCCAGGTGGTGAT
**Glutathione peroxide 1**	60 °C – 104 bp
Forward 5′ – 3′	GCTGGTGGTGCTGGGCTTCC
Reverse 5′ – 3′	ACCCTTTTCCTGGACGAACGTACT

## Results and Discussion

### Array expression data

**Figure 1:**
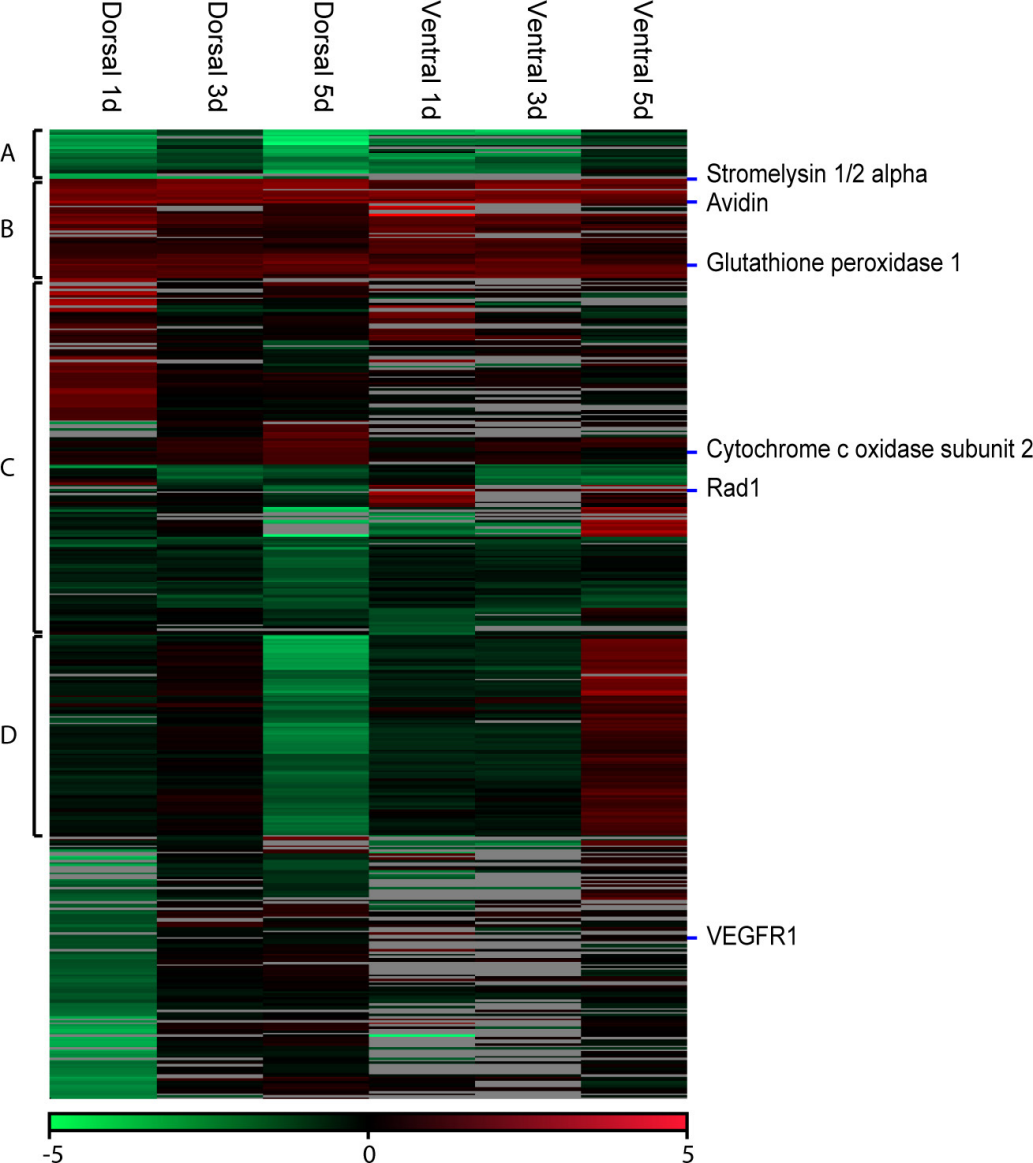
Heat map of expression patterns derived from the microarrays. The heat map is subdivided into four clusters depending on the expression patterns. The location of the genes used for "quantitative real-time (qRT)-PCR analysis is shown on the heat map. Only contigs with a minimum number of two valid values have been selected for the heat map. For the heat map, we took 467 candidates into account, but this number was reduced to 465 candidates by filtering for valid values. Within the clusters, we had 23 transcripts within cluster A, 48 transcripts within Cluster B, 171 transcripts within Cluster C, and 94 transcripts within Cluster D.

**Figure 2:**
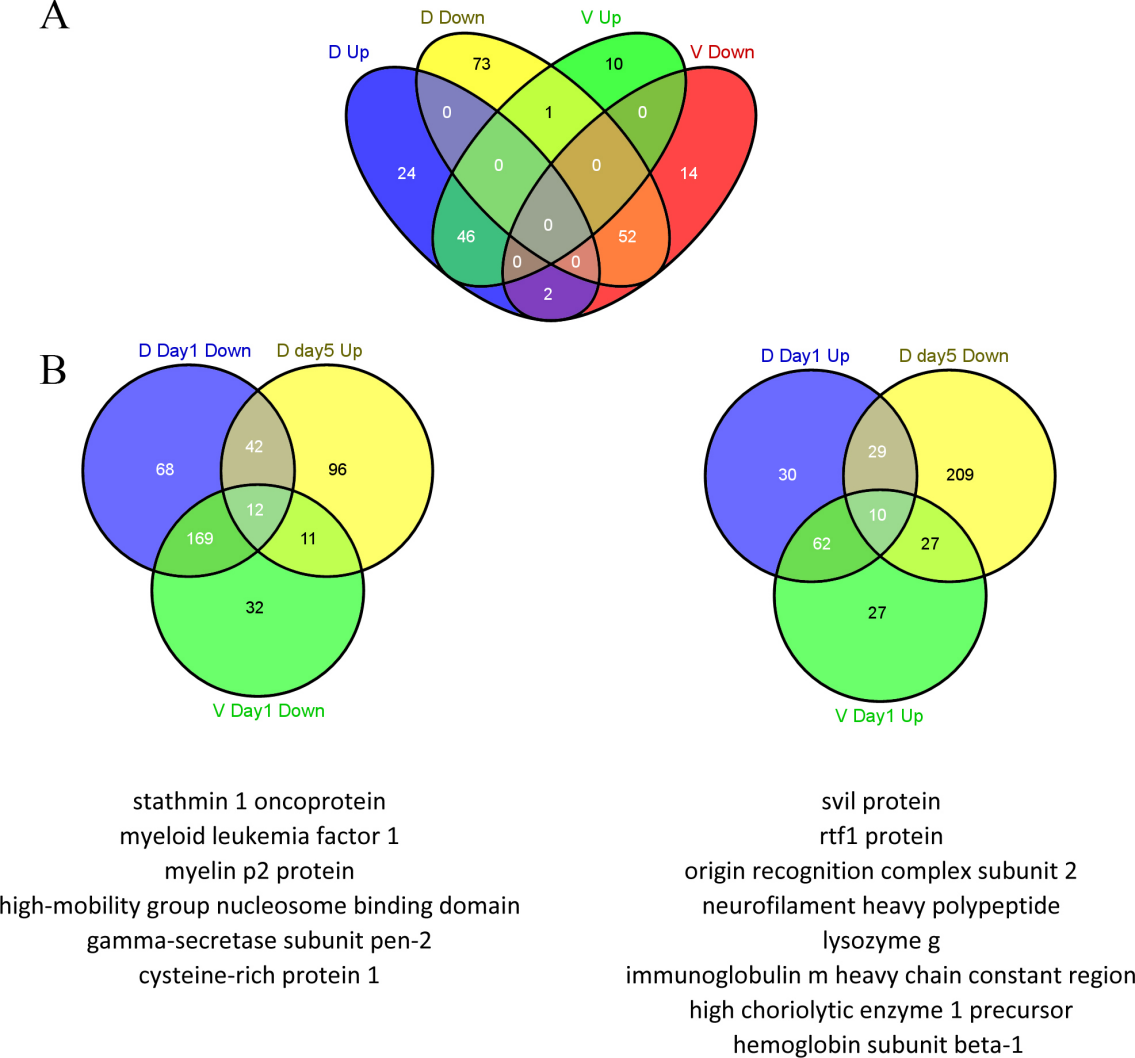
Expression comparison among dorsal/ventral iris in selected time points. **A**: Venn diagram for contigs consistently up- or downregulated in dorsal or ventral iris during all the time points. D up: Contigs upregulated in the dorsal iris during all the time points. D down: Contigs downregulated in the dorsal iris during all the time points. V up: Contigs upregulated in the ventral iris during all the time points. V down: Contigs downregulated in the ventral iris during all the time points. **B**: Venn diagrams for contigs that are downregulated in the dorsal iris and the ventral iris at day 1, and upregulated in the dorsal iris at day 5, and the opposite. Annotated genes are included below each Venn graph. D: dorsal iris; V: ventral iris.

Microarray analysis obtained 804 spots with differential expression 1, 3, or 5 days post-lentectomy in the dorsal or ventral iris. Combining the expression values of the replicates yielded 467 different sequences, which we refer to as contigs. Appendix 1 contains the list of all assembled contigs, their annotation, expression in the microarrays, and an identifier that can be used to retrieve more information from the newtomics database. Differentially regulated contigs were clustered and visualized with a heat map (Figure 1). A general pattern that emerges from a visual inspection of the heat map is that there is common up- or downregulation, when compared with the intact iris (0 day) in the dorsal and ventral iris (see clusters A and B and part of Cluster C, Figure 1). In other words, genes that show upregulation during 1, 3, and 5 days post-lentectomy in the dorsal iris show the same differential expression in the ventral iris (Cluster B, Figure 1). Reversely, this is the case for several downregulated contigs, too (Cluster A, Figure 1). In addition, Figure 2 presents a direct comparison of dorsal and ventral genes that are consistently up- or downregulated without regarding single time points. Forty-six contigs out of 72 (63.9%) and 46 out of 57 (80.7%) are commonly upregulated in the dorsal and ventral iris, respectively. Fifty-two contigs out of 126 (41.3%) and 52 out of 68 (76.5%) are commonly downregulated in the dorsal and ventral iris, respectively. These results strengthen the hypothesis that dorsal and ventral irises initiate the same first steps of lens regeneration. In a different comparison, we examined which genes are regulated at dorsal day 5 compared with day 1 (D day 1, V day 1 downregulation versus D day 5 upregulation and opposite). This analysis could potentially reveal genes related to transdifferentiation. Interestingly, among regulated genes we found cytoskeletal organization-related proteins (stathmin 1 [17], svil protein [18]) and cell pluripotency-maintenance factor (rtf1 [19]; Figure 2B).

In addition to the clusters, we identified another remarkable pattern. This pattern is defined by an inversely regulated time point (five days; Cluster D; Figure 1). Other time points are not regulated. This cluster consists of 94 non-redundant members. When we examined this cluster for potential enrichment of assigned function, we found only 14 candidates with a similarity to public available sequences. However, most of the candidates lack counterparts in higher vertebrates, even as the corresponding sequences have a sufficient sequence length (see Table 2). Within the annotated contigs, we found an isoform of suppression of tumorigenicity 7, known to play a crucial role in tumor suppression through regulating genes involved in maintaining the cellular structure [20] or remodeling the extracellular matrix [21]. Altogether this indicates that Cluster D might represent newt specific genes that are inversely regulated in the dorsal and ventral iris 5 days after lentectomy.

**Table 2:** List of annotated contigs found in cluster D with sufficient sequence length.

Contigname	Accession	Description	Organism	Score	E value	Length
Contig_122	ADJ80991.1	CR1–2	Lycodichthys dearborni	90.5	1e-17	638
Contig_180	NP_001006347.1	OTU domain-containing protein 6B	Gallus gallus	152	4e-42	493
Contig_185	EGV97740.1	Pol polyprotein	Cricetulus griseus	75.5	4e-15	381
Contig_187	XP_002737624.1	PREDICTED: polyprotein-like	Saccoglossus kowalevskii	128	5e-30	755
Contig_2	ACJ43736.1	NADH dehydrogenase subunit 5	Notophthalmus viridescens	390	6e-129	863
Contig_207	XP_003216825.1	PREDICTED: transmembrane protein 205-like	Anolis carolinensis	230	6e-73	672
Contig_215	XP_002942833.1	PREDICTED: androgen-induced gene 1 protein-like	Xenopus (Silurana) tropicalis	201	8e-61	761
Contig_258	ACO09746.1	Lysozyme g	Osmerus mordax	238	2e-76	653
Contig_277	ABI93642.1	suppression of tumorigenicity 7, isoform a, 5 prime	Neofelis nebulosa	330	3e-110	808
Contig_287	XP_003221516.1	PREDICTED: 60S ribosomal protein L9-like	Anolis carolinensis	248	4e-81	454
Contig_302	XP_418054.2	PREDICTED: prolargin	Gallus gallus	271	5e-86	791
Contig_34	XP_002193668.1	PREDICTED: eukaryotic translation initiation factor 2B, subunit 3 gamma	Taeniopygia guttata	325	6e-106	761
Contig_399	NP_990623.1	GTPase HRas precursor	Gallus gallus	338	4e-115	721
Contig_46	BAE25396.1	unnamed protein product	Mus musculus	122	3e-28	1106

### Functional annotation

Of the 467 contigs, 265 have a significant sequence similarity to public available proteins, whereas 192 have assigned GO terms. We further investigated these contigs and identified that the most interesting functional annotation, in concordance with other regeneration model systems, is related to cell cycle, proliferation, extracellular region, redox homeostasis, mitochondrion, DNA repair, and parental GO terms. The list of GO annotated contigs is shown in Appendix 2.

### Cell cycle reentry, proliferation, and DNA repair

**Figure 3:**
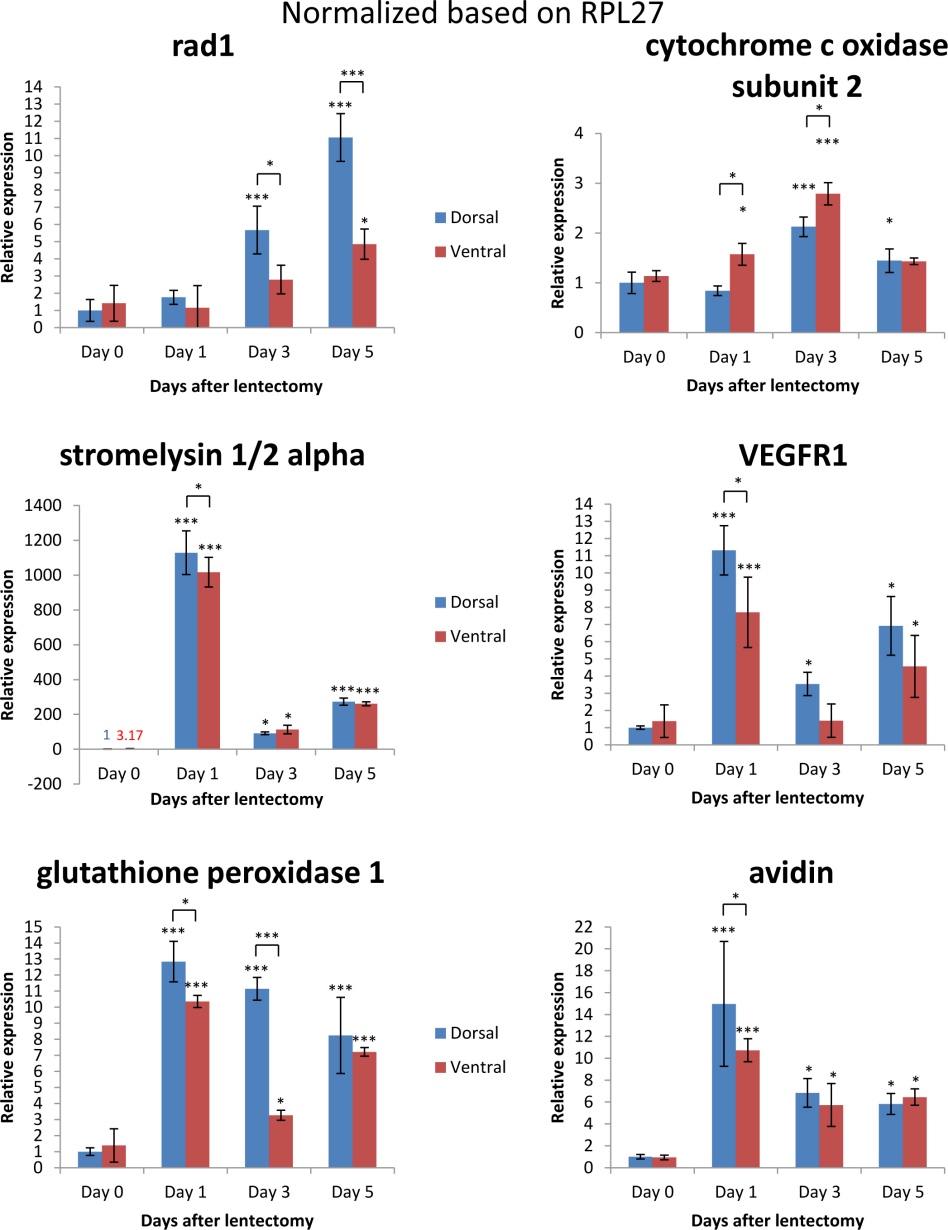
Relative expression of rad1, cytochrome oxidase subunit 2, stromelysin 1/2 alpha, vascular endothelial growth factor receptor 1 (VEGFR1), glutathione peroxidase 1, and avidin genes after quantitative real-time (qRT)-PCR 0, 1, 3, and 5 days after lentectomy for dorsal and ventral pigmented epithelial cells (PECs) normalized with housekeeping gene ribosomal protein large 27 (RPL27). One asterisk (*) indicates p value smaller than 0.05 (p<0.05). Three asterisks (***) indicate p value smaller than 0.001 (p<0.001). Asterisk located above the expression columns indicates significance between the sample at a particular time point and the intact iris. Asterisk located above the black line indicates significance between the samples of the same day. The statistical analysis used is the Student *t* test. Bars on graph indicate standard deviation.

Previous studies revealed that by day 4 post-lentectomy the dorsal and ventral irises reenter the cell cycle [22]. We found that bccip homolog and myeloid leukemia factor 1, two proteins that cause cell cycle arrest, were slightly downregulated 3 days after lentectomy and then were upregulated 5 days after lentectomy in the dorsal iris. In the ventral and dorsal iris in all time points, peroxiredoxin 1, a protein that has been shown to play a role in proliferation and to be expressed in melanosomes, was upregulated (Table 3) [23,24]. In addition, tmsb4, also known as thymosin beta 4, was upregulated in all time points in the ventral and dorsal iris. This protein is known for actin organization and the S phase entry of the cell cycle. Among the DNA repair and proliferation factors, we found expression of rad1 and vascular endothelial growth factor receptor 1 (VEGFR1). Rad1 is a protein that plays a role in DNA repair of double-strand breaks and is part of the 9.1.1 complex with Rad9 and Hus1 [25]. VEGFR1 is a receptor that is important in the proliferation of endothelial cells, angiogenesis, and cancer. qRT-PCR analysis demonstrated Rad1 was upregulated in the dorsal iris 3 days (p<0.001) and 5 days (p<0.001) compared to 0 day post-lentectomy. Further, rad1 was upregulated in the ventral iris only 5 days (p<0.05) compared to 0 day after lentectomy. In addition, the dorsal iris showed upregulation of rad1 compared to the ventral iris 3 days (p<0.05) and 5 days (p<0.001) post-lentectomy (Figure 3). The other candidate, VEGFR1, was upregulated in the ventral and dorsal iris 1 day (p<0.001) and 5 days (p<0.05) compared to 0 day post-lentectomy. Overall, during regeneration, the dorsal iris has a higher VEGFR1 expression level compared to the ventral iris. These results are consistent with the onset of proliferation during the early phases of lens regeneration and are in context with the robustness in DNA repair inferred during regenerative activities. For example, Eguchi et al. showed that lens regeneration is not affected after repeated lentectomies (18 times over 16 years) in the Japanese newt, *Cynops pyrrhogaster* [26].

**Table 3:** Selected contigs related to cell cycle, proliferation and DNA repair. Values are log_2_fc

Function	Contig Annotation	Dorsal Day 1	Dorsal Day 3	Dorsal Day 5	Ventral Day 1	Ventral Day 3	Ventral Day 5
Cell cycle, proliferation and DNA repair	bccip homolog	-	−0.17	1.60	-	-	-
	myeloid leukemia factor 1	−1.63	−0.12	0.13	−0.14	-	-
	peroxiredoxin-1	2.13	1.85	2.65	2.02	2.09	1.78
	vascular endothelial growth factor receptor 1	−1.31	-	−0.18	-	-	0.14
	26s proteasome complex subunit dss1	−0.83	0.55	−4.14	−2.42	−2.14	0.10
	tmsb4×protein	1.11	1.62	1.76	0.83	1.49	0.93
	tubulin beta 6	1.86	2.32	1.90	2.50	1.89	1.81
	cell cycle checkpoint protein rad1-like	−1.42	−0.12	−1.77	-	0.95	2.07
	cyclin-dependent kinase 7	-	-	−0.12	−1.82	-	-

We think that these patterns reflect the preparation of the iris day 1 post-lentectomy to initiate reentry in the cell cycle to proliferate. This was accompanied by DNA repair factors that will most likely be activated to maintain the integrity of DNA especially in the dorsal iris (twofold upregulation of rad1 in dorsal versus ventral, Figure 3). These findings support the thesis that the dorsal iris prepares more rigorously for regenerating the lens.

### Reactive oxygen species and mitochondria-related proteins

As shown in Table 4, enzymes related to redox homeostasis are upregulated in all time points of the ventral and the dorsal iris. Glutathione peroxidase 1, peroxiredoxin mitochondrial-like (peroxiredoxin 3), sh3 domain-binding glutamic acid-rich-like protein 3 (SH3BGRL3), and thioredoxin are proteins responsible for reducing reactive oxygen species (ROS) to protect the cells from ROS-related stress and apoptosis. We confirmed these findings derived from the microarray data with qRT-PCR of glutathione peroxidase 1 (Gpx1). Gpx1 was upregulated 1, 3, and 5 days after lentectomy in the dorsal (p<0.001 for all days) and ventral iris (p<0.001 for 1 and 5 days, p<0.05 for 3 days) compared to the intact iris. Gpx1 was upregulated in the dorsal iris 1 day (p<0.05) and 3 days (p<0.001) compared to the ventral iris (Figure 3). Peroxiredoxin 6, which was downregulated, seems to be linked with the redox system the cells chose to reduce ROS.

**Table 4:** Selected contigs related redox homeostasis and mitochondria. Values are log_2_fc

Function	Contig Annotation	Dorsal Day 1	Dorsal Day 3	Dorsal Day 5	Ventral Day 1	Ventral Day 3	Ventral Day 5
redox homeostasis	glutathione peroxidase 1	2.21	1.96	1.40	1.82	1.74	1.29
	peroxiredoxin- mitochondrial-like	0.85	0.49	0.28	1.69	1.04	0.14
	peroxiredoxin-1	2.13	1.85	2.65	2.02	2.09	1.78
	sh3 domain-binding glutamic acid-rich-like protein 3	0.39	0.86	1.55	0.56	0.96	0.78
	thioredoxin	1.38	0.74	0.37	0.80	0.39	0.12
	redox-regulatory protein fam213a isoform 1 precursor	-	0.45	1.47	0.29	-	0.31
	peroxiredoxin 6	−0.27	−0.72	−1.39	−0.35	−0.71	−0.51
							
mitochondria	a chain crystal structures of transition state analog inhibitors of inosine monophosphate cyclohydrolase	−2.11	−1.06	−2.08	−2.08	−1.42	−0.52
	acetyl- mitochondrial precursor	−0.33	-	−0.65	−1.34	−1.33	−0.61
	adp atp translocase 1	−1.39	−0.33	−0.30	−0.67	−0.64	−0.09
	isocitrate dehydrogenase	−0.47	−1.56	−1.40	−0.41	−1.83	−1.71
	succinate dehydrogenase	−2.54	-	−0.11	−0.23	−0.30	-
	cytochrome b-c1 complex subunit mitochondrial-like	−2.59	0.24	0.42	0.30	0.29	0.11
	cytochrome c oxidase subunit 2	1.09	0.62	1.08	−0.51	0.38	−0.37
	cytochrome c oxidase subunit 3	-	0.42	1.81	-	0.80	-
	cytochrome c oxidase subunit iv isoform 1	0.33	0.64	1.34	0.18	0.56	−0.15
	cytochrome testis-specific	1.12	1.20	0.53	0.55	1.30	0.88

Another obvious pattern was formed by mitochondrial enzymes related to ketone metabolism, ATP synthesis, and purine biosynthesis that are downregulated in the dorsal and ventral iris in all investigated time points. However, the cytochrome c oxidases were mostly upregulated. Cytochrome c oxidase subunit 2 is a mitochondrial component involved in oxidative phosphorylation. Dysregulation of this gene is mostly due to mitochondrial-related stress [27]. Since lentectomy is a surgical procedure, it naturally creates stress. Cytochrome c oxidase subunit 2 was upregulated in the ventral iris 1 day (p<0.05) and 3 days (p<0.001) compared to 0 day post-lentectomy (qRT-PCR validation, Figure 3). Cytochrome c oxidase subunit 2 was also upregulated in the dorsal iris 3 days (p<0.001) and 5 days (p<0.05) compared to 0 day post-lentectomy. In addition to these common regulations, there was an inverse differential expression between the dorsal and ventral iris at day 1 (p<0.05) and 3 (p<0.05) with increased expression levels in the ventral iris.

Additionally, glutathione peroxidase 1 and cytochrome oxidase subunit 2 had opposite differential expression between the dorsal and ventral irises 1 and 3 days post-lentectomy. *Gpx1* was upregulated in the dorsal iris after 1 day (p<0.05) and 3 days (p<0.001) post-lentectomy compared to the ventral iris. Cytochrome c oxidase subunit 2 was upregulated in the ventral iris 1 day (p<0.05) and 3 days (p<0.05) post-lentectomy compared to the dorsal iris. Both genes returned to the same expression levels 5 days post-lentectomy (p>0.05; Figure 3). These two genes are connected via ROS. Stress caused by ROS can affect mitochondria in which Gpx1 can reduce ROS [28]. The high expression level of *gpx1* in the dorsal iris has a potential role in reducing ROS faster than the ventral iris, affecting the rate of cell activation and responsiveness in early stages of regeneration.

To sum up, these results indicate that stress caused by ROS is answered through upregulating of enzymes that reduce ROS in the iris, thus protecting the cells. ROS might cause mitochondrial stress, which is reflected by downregulation of certain mitochondrial enzymes. It is not yet clear whether ROS is a signal of regeneration or ROS is produced by the surgical operation through cutting the cornea. However, these patterns provide an impetus for further investigation.

### Extracellular matrix and transdifferentiation

Another group of annotated proteins can be described as extracellular matrix–related proteins (Table 5). Most of these proteins are upregulated in the dorsal and ventral iris in all time points. For example, clusterin is known to play a role in extracellular matrix organization by acting as a stress-induced secreted chaperone protein and having the ability to inhibit stress-induced protein precipitation [29]. Mmp18 and stromelysin 1/2 alpha are matrix metalloproteinases that degrade certain types of matrix, including collagen III, IV, IX, and X. Their role is critical for transdifferentiation since extracellular matrix and remodeling proteins are needed for cell fate change. qRT-PCR validation of stromelysin 1/2 alpha confirmed upregulation in the dorsal and ventral irises 1 day (p<0.001 for both), 3 days (p<0.05 for both), and 5 days (p<0.001 for both) compared to 0 day after lentectomy. There was a slight upregulation of stromelysin 1/2 alpha in the dorsal iris compared to the ventral iris at day 1 post-lentectomy (p<0.05). The high expression of stromelysin 1/2 alpha is consistent with other studies in which it was upregulated in the dorsal iris and the ventral iris 8 days post-lentectomy compared to the intact iris [6]. The results allow us to assume that the process of tissue remodeling starts immediately after lentectomy since the matrix metalloproteinases were upregulated 1,200-fold at 1 day post-lentectomy in the ventral and dorsal irises (p<0.001). In the context of highly increased expression levels of matrix metalloproteinases, collagen I was downregulated, a result consistent with the fact that the cells degraded the collagen around them for later proliferation. Another protein known to be located in the extracellular matrix is avidin. qRT-PCR confirmed the expression data of our microarrays and showed upregulation in all investigated days after lentectomy for the ventral and dorsal irises compared to the intact iris. Avidin is thought to have an impact as a defense protein against microbial infections that could be caused through the surgery process [30].

**Table 5:** Selected contigs related to extracellular matrix. Values are log_2_fc

Function	Contig Annotation	Dorsal Day 1	Dorsal Day 3	Dorsal Day 5	Ventral Day 1	Ventral Day 3	Ventral Day 5
Extracellular matrix	clusterin precursor	1.66	1.96	2.64	1.26	2.06	2.23
	loc100036815 protein / interleukin-8	0.59	0.30	0.84	1.75	1.64	0.91
	mmp18 protein	1.56	2.38	2.24	-	-	2.45
	stromelysin-1 2-a	−0.14	-	-	3.43	-	-
	collagen type i alpha 1	0.06	−2.09	−1.20	−0.27	−2.37	−2.59
	d chain crystal structure of xenavidin	3.09	2.6	2.67	3.13	2.89	1.54

### Concluding remarks

With this report, we further expand our knowledge of gene expression during early lens regeneration in *Notophthalmus viridescens*. Our results clearly indicate that during early stages there are not many qualitative differences between the dorsal iris and the ventral iris. Rather, quantitative differences might be critical for the dorsal and ventral iris’s contribution in lens regeneration. The lack of gene annotation for a group of potential newt/amphibian specific genes points toward the hypothesis that some regeneration-related proteins might be evolutionarily conserved only in amphibians. However, certain signatures for regeneration have been verified. Cell proliferation was combined with a robust quality check mechanism to keep the DNA intact and to be passed to new cells without mutations. We also report that the iris employs enzymes to reduce ROS. Both homeostasis mechanisms were upregulated on the site where the regeneration process occurs, the dorsal iris. In addition, as soon as 1 day after lentectomy, cells started to prepare for the transdifferentiation by upregulation of certain matrix metalloproteinases and regulation of their extracellular matrix, a step crucial to the upcoming differentiation to lens cells. As more studies on global gene expression become available, certain differences might lead to elucidating the much-coveted mechanisms of regenerative processes.

## Appendix 1. Array expression data.

To access the data, click or select the words “Appendix 1.” This will initiate the download of a compressed (pdf) archive that contains the file.

## Appendix 2. Functional annotation.

To access the data, click or select the words “Appendix 2.” This will initiate the download of a compressed (pdf) archive that contains the file.
